# Prevalence and factors influencing reporting of true periodontal chief complaints: A retrospective analysis

**DOI:** 10.1002/cre2.385

**Published:** 2020-12-22

**Authors:** Ali A. Abdulkareem, Nada K. Imran, Rukhosh H. Abdulraheam, Sarhang S. Gul

**Affiliations:** ^1^ Department of Periodontics, College of Dentistry University of Baghdad Baghdad Iraq; ^2^ Basic Science Department, College of Dentistry University of Sulaimani Sulaymaniyah Iraq; ^3^ Periodontics Department, College of Dentistry University of Sulaimani Sulaymaniyah Iraq

**Keywords:** periodontal disease, prevalence, retrospective, true chief complaint

## Abstract

**Objectives:**

To investigate the prevalence of true periodontal chief complaints (CC) and the factors affecting their reporting by patients with periodontal diseases (PD).

**Materials and Methods:**

This cross‐sectional study was based on retrospective analysis of available periodontal records. Different personal and demographic variables were obtained from these records including CC, age, gender, working status, past medical/dental history, smoking status and diagnosis. In addition, clinical parameters of plaque index, gingival index, probing pocket depth (PPD), and number of missing teeth. Periodontal CC were retrieved and divided either into true periodontal (bleeding, tooth mobility, and alteration in gingival color/shape) or others (emergency and esthetic‐related) CC.

**Results:**

A total of 1161 records were included in the final analysis. Results showed that only 287 (24.7%) of patients reported true periodontal CC whereas the remaining 874 (75.3%) patients were not aware about symptoms of PD. Regression modeling indicated that reporting of true CC was positively associated with smoking and PPD but negatively associated with number of missing teeth and gender (male).

**Conclusions:**

Results suggested that recognition of true periodontal CC by the patients was low. Reporting of true periodontal CC was significantly associated with smoking, PPD, female and lower number of missing teeth. These results shed light on the importance of increasing public knowledge about PD which is essential to aid people in recognizing these diseases at early stages.

## 1 INTRODUCTION

Periodontal diseases (PD) are multifactorial, multi‐microbial diseases that progressively damage the supporting structure of the teeth. Dental biofilm is considered as a major cause of these diseases; however, the majority of destruction happens as a result of host immune response (Kinane et al., 2017). Despite diversity of PD and conditions; yet, the most common forms affecting a large portion of the populations worldwide are gingivitis and periodontitis and the latter is considered among the leading causes of tooth loss in adults (Dye, 2012; Kassebaum et al., 2014). Clinically, PD can be diagnosed based on the signs and symptoms such as biofilm deposition on teeth surfaces, bleeding on probing and/or brushing, gingival recession, formation of periodontal pockets, clinical attachment loss (CAL), halitosis, and tooth mobility (Chapple, 2009; Tonetti et al., 2018). It is important to acknowledge that due to their slowly progressing nature, PD are painless; however, pain can be experienced during acute necrotizing PD, periodontal abscess and traumatic occlusion (Gaurilcikaite et al., 2017).

Like any other diseases, the diagnosis of PD relies upon keeping a patient information record that includes patients' chief complaint (CC), medical and dental history, followed by clinical and radiograph examinations (Brunsvold et al., 1999). Patients' CC and reasons for seeking care are valuable sources of information for clinicians. Compared with other presenting symptoms, attention to the CC can guide admitting triage, diagnosis, and early treatment. In general, the majority of patients seeking periodontal treatment complain of bleeding (on brushing or spontaneous), halitosis, unpleasant esthetic, elongated teeth (gingival recession) and mobility (Brunsvold et al., 1999; Yeh & Lai, 2011). However, these problems can be associated with other oral diseases such as dental caries and oral mucosal lesions or with systemic diseases beyond the oral cavity (Bollen & Beikler, 2012).

Negligence of PD‐associated symptoms is an internationally reported problem mostly attributed to the lack of public awareness about etiology, signs and symptoms of PD (Hosadurga et al., 2015; Lung et al., 2005; Luo & Wu, 2017). In fact, this is not merely due to deficiency of knowledge but also to the apparent impact of media. The latter significantly increased the public obsession about having a perfect pearly smile and Hollywood stars' teeth in the last‐decades (Poon, 2018; Theobald et al., 2006). This media influence on esthetic aspects of dentistry contrasts with the lesser attention paid to motivational/educational programs about alarming symptoms of PD and the preventive approaches (Dumitrescu, 2016).

Expression of true periodontal CC by the patient is of paramount importance for the dentist to achieve proper diagnosis, prevention and treatment of PD (Yeh & Lai, 2011). Therefore, this study aimed to investigate the prevalence together with the factors that influence reporting of true periodontal CC of PD by patients seeking dental treatment.

## 2 MATERIALS AND METHODS

### 2.1 Study design

This study was a retrospective cross‐sectional analysis of the periodontal records of patients attending the College of Dentistry, University of Baghdad from 2015 to 2018. Permission to access the data was obtained from the scientific committee of the Department of Periodontics/College of Dentistry/University of Baghdad (Ref 85 in 09/10/2018) following guidelines of the Helsinki declaration for human studies.

### 2.2 Study population and outcomes

The total number of available periodontal records for the aforementioned period was 1865 which were considered on the basis of the following criteria:

- Age (≥18 years).
- Patients seeking periodontal treatment for the first time.
- Diagnosed with gingivitis or periodontitis according to 1999 classification (Armitage, 1999). Briefly, Gingivitis was defined by probing pocket depth (PPD) <4 mm with signs of inflammation. While periodontitis was defined as ≥2 interproximal sites with PPD ≥4 mm (not on same tooth) or one site with PPD ≥5 mm (Eke et al., 2012). While the new classification of periodontal disease and conditions clearly defined periodontal health as a separate entity (bleeding on probing [BOP] < 10%) and gingivitis (BOP≥10%; Caton et al., 2018). The older version of the classification did not consider using a clear definition to discriminate periodontal health from gingivitis. Therefore, misdiagnosis between mild and localized plaque‐induced gingivitis and healthy cases was expected which could represent a potential bias for this study.

Any record not meeting the above‐mentioned criteria was excluded. After sorting the records according to the inclusion/exclusion criteria, information for each patient was collected and recorded on a spreadsheet using Microsoft excel software (version 2016, Microsoft Corporation, Redmond, WA). Personal and demographic information including CC, age, gender, past medical (such as diabetes)/dental history, smoking status (yes or no), working status and diagnosis. This was followed by collection of clinical parameters, using Williams periodontal probe with 1‐2‐3‐5‐7‐8‐9‐10 mm marking, including plaque index (PI; Silness & Loe, 1964), gingival index (GI; Loe & Silness, 1963), PPD, and number of missing teeth.

Patients' periodontal CC were divided into true and other CC. True periodontal CC included gingival bleeding (spontaneous or upon stimulation), mobility of the teeth, and alteration in gingival shape/morphology (enlargement and/or recession; Brunsvold et al., 1999; Elhassan et al., 2017), whereas other CC included referral, emergency and esthetic‐related reasons not necessarily caused as sequelae of PD.

The primary outcomes were to determine the prevalence of true periodontal CC expressed by the patients (dependent variable) and their degree of association with personal/demographic variables and clinical parameters (independent variables).

### 2.3 Statistical analysis

Descriptive statistics, including frequency/percent, mean, and standard deviation, were used and expressed in pie charts, tables, and histograms. Intergroup comparison of clinical parameters between patients expressing true periodontal CC and those who did not was performed by using unpaired t‐test. Stepwise logistic regression modeling was used to determine the association between the dependent binary variable (true periodontal CC: 1, other CC: 0) and different independent variables of the study. All statistical analyses were performed by using SPSS software (Version 25, IBM, NY). Differences were considered significant at *p* < 0.05.

### 2.4 Ethics statement

The Ethics Committee of College of Dentistry, University of Baghdad approved the protocol of the study, which proceeded in accordance with the Declaration of Helsinki for human researches.

## 3 RESULTS

**FIGURE 1:**
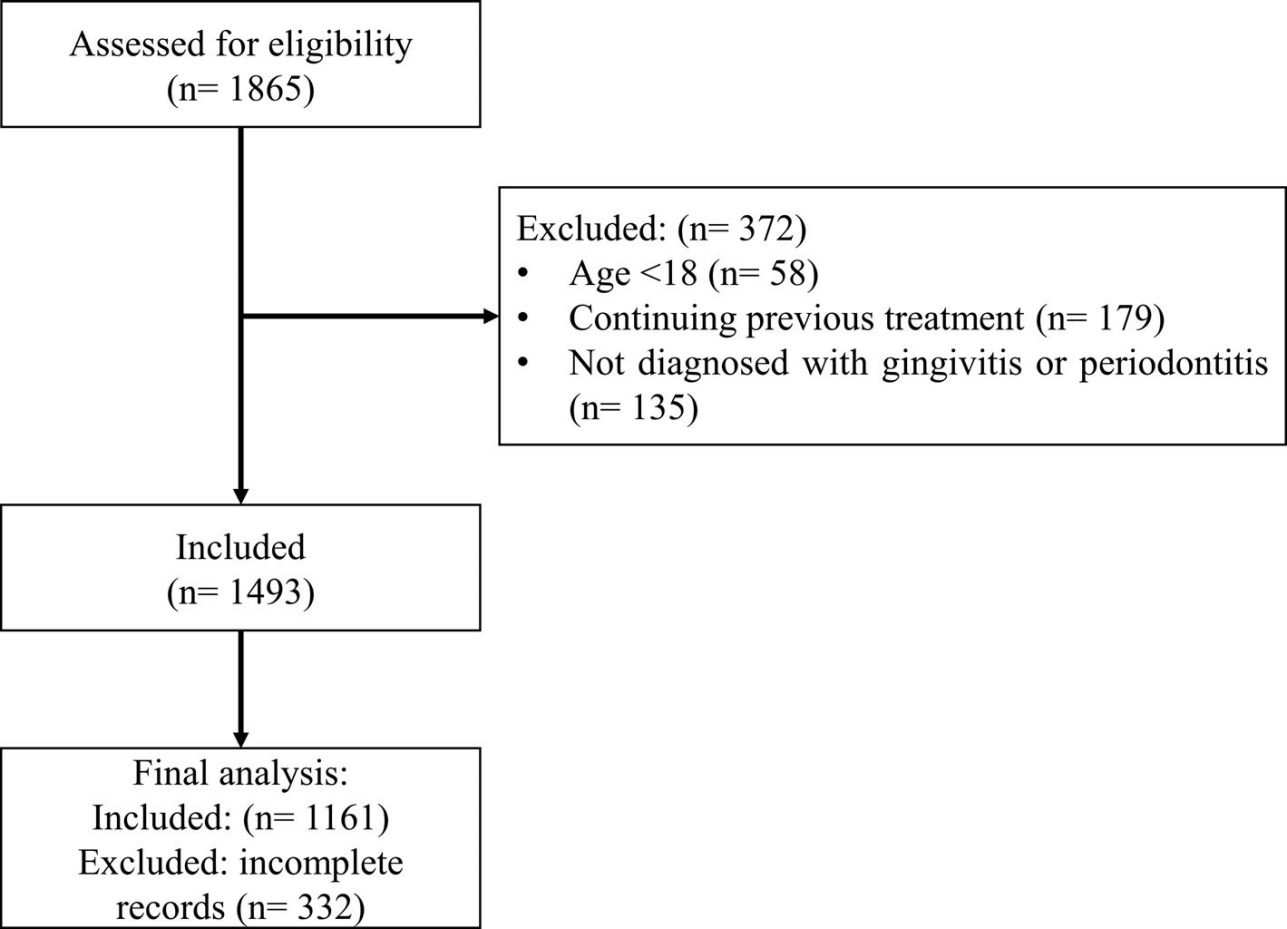
Flow diagram of the study

According to inclusion/exclusion criteria, a total of 372 records (out of 1865) were excluded. Later, another 332 records were excluded for being incomplete leaving 1161 for the final analysis (Figure 1). The mean age of the study population was 42.48 ± 13.80 years, ranging from 18 to 76 years old. The distribution of the recorded CC, whether true periodontal CC or not, according to the different patient characteristics, is illustrated in Table 1. Results showed that the CC of 874 (75.3%) patients were not related to PD and only 287 (24.7%) patients who reported CC expressed true periodontal symptom(s) (Table 1).

**TABLE 1:** Distribution of chief complaints according to different independent variables

Variables	True periodontal CC^a^	Other CC^a^	Total^a^
*Gender*			
Male	165 (21.5)	601 (78.5)	766 (34)
Female	122 (30.9)	273 (69.1)	395 (66)
*Age (years)*			
<43	155 (27.6)	407 (72.4)	562 (48.4)
≥43	132 (22.1)	467 (77.9)	599 (51.6)
*Systemic disease*			
Yes	57 (23.5)	185 (76.5)	242 (20.8)
No	230 (25.1)	689 (74.9)	919 (79.2)
*Past dental history*			
Yes	120 (26.3)	337 (73.7)	457 (39.4)
No	167 (23.8)	537 (76.2)	704 (60.6)
*Smoking status*			
Yes	55 (16.5)	279 (83.5)	334 (28.8)
No	232 (28.1)	595 (71.9)	827 (71.2)
*Diagnosis*			
Gingivitis	59 (27.6)	155 (72.4)	214 (18.4)
Periodontitis	228 (24.1)	719 (75.9)	947 (81.6)
*Work status*			
Employed	144 (21.4)	528 (78.6)	672 (57.8)
Unemployed	120 (30.0)	280 (70)	400 (34.5)
Retired	23 (25.8)	66 (74.2)	89 (7.7)
*Total*	287 (24.7)	874 (75.3)	1161 (100)

^a^ Frequency (percentage).

**FIGURE 2:**
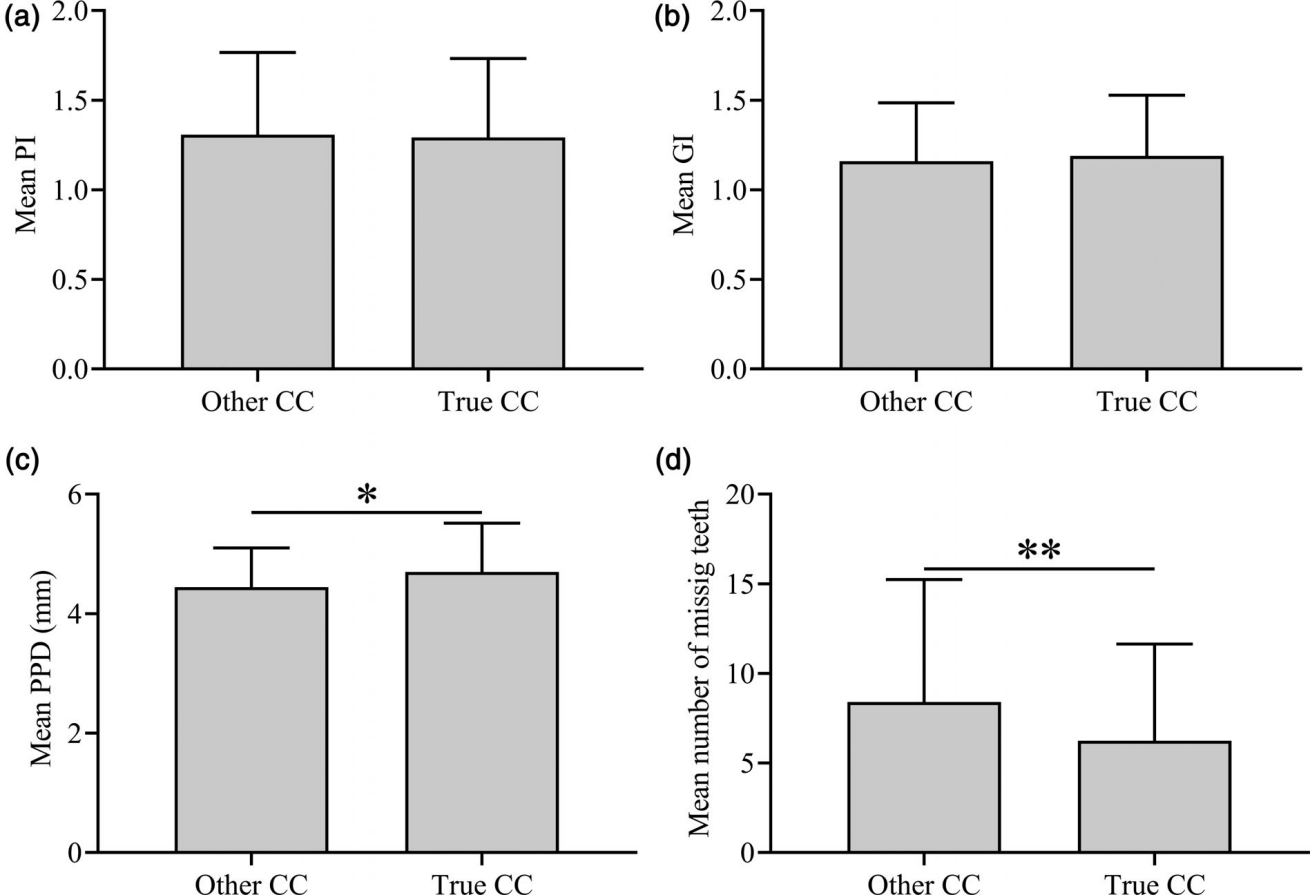
Comparison of PI, GI, PPD and missing teeth between subjects with true periodontal chief complaints (CC) and other chief complaints. For PI and GI, no significant differences were observed between the two groups (a and b). However, patients reporting true periodontal CC exhibited significantly deeper periodontal pockets than those reporting other CC (c). The opposite was observed in relation to numbers of missing teeth which were significantly higher in patients reporting other CC as compared to those expressing true periodontal disease CC (d). *p < 0.05, **p < 0.001

Analysis of the clinical parameters between the two CC groups showed no significant difference between these groups in relation to PI and GI (Figure 2a,b). However, PPD was significantly higher (*p* < 0.05) in the true periodontal CC group than among those who did not express true periodontal CC of PD (Figure 2c). On the other hand, number of missing teeth was significantly higher (*p* < 0.001) in patients not reporting true periodontal CC as compared to those who did (Figure 2d).

Logistic regression modeling was used to determine the association between independent variables and reporting of the true periodontal disease CC (dependent variable). Stepwise method was used to exclude independent variables that did not have significant impact on patients' reporting of true periodontal CC. All independent variables were excluded except PPD, missing teeth, male, and smoking that showed 75.3% of certainty to predict true periodontal CC (Table 2). Both smoking and increasing PPD showed a positive and significant association (OR 1.7 and 1.142, respectively) with true periodontal CC (Table 3), while negative significant associations were observed for number of missing teeth (OR 0.938) and gender (male) (OR 0.682) with the true periodontal CC (Table 3).

**TABLE 2:** Stepwise regression model to predict true periodontal chief complaint as dependent variable

	Observed		Predicted		
			Chief complaints		Percentage correct
			Other	True	
Step 1	All variables	Others	868	5	99.4
		True	283	4	1.4
	Overall percentage				75.2
Backward stepwise	PPD, missing teeth, Gender (male), smoking	Others	869	4	99.5
		True	282	5	1.7
	Overall percentage				75.3

**TABLE 3:** Logistic regression for each independent variable for reporting true periodontal chief complaint

	B	S.E.	Wald	Exp (B)	95% CI	*p*‐value*
Diagnosis	0.01	0.187	0.003	1.01	0.7–1.4	0.958
Systemic condition	0.037	0.181	0.042	1.037	0.7–1.4	0.840
Past dental history	−0.056	0.144	0.151	0.946	0.07–1.2	0.698
GI	0.149	0.216	0.476	1.160	0.76–1.77	0.490
PI	0.204	0.161	1.620	1.227	0.82–1.66	0.203
Occupation	−0.484	0.278	3.027	0.616	0.34–1.04	0.082
PPD	0.121	0.045	0.3473	1.142	1.03–1.23	**0.002**
Missing teeth	−0.064	0.012	26.615	0.938	0.906–0.956	**0.0001**
Gender (male)	−0.382	0.154	6.177	0.682	0.38–0.98	**0.013**
Smoking	0.531	0.181	8.601	1.700	1.19–2.46	**0.003**

* Significance at *p* <0.05.

## 4 DISCUSSION

True periodontal CC were reported by less than 25% of the patients. According to the regression model, recognition of true PD symptom(s) was significantly correlated with PPD, missing teeth, gender (male), and smoking. This study was performed to investigate the prevalence of true periodontal CC reporting by patients and the factors affecting their expression.

Interestingly, in one study, over 50% of patients affected by periodontitis reported referral and desire to save teeth as the main CC without knowing of the existence of PD (Brunsvold et al., 1999). Meanwhile, in the same study, true periodontitis symptoms such as bleeding ranked in third place, reported only by 20% of PD patients. Additionally, mobility of teeth was only reported by 9% of the patients (Brunsvold et al., 1999), confirming that perceptible tooth mobility is usually a symptom of severe and late stages of periodontitis (Coventry et al., 2000). Another study likewise reported referral by members of the dental health team as the main CC of patients (32%) affected by moderate to severe periodontitis, whereas true periodontal CC were not well‐recognized (Elhassan et al., 2017). These results follow a similar pattern to our findings which showed that less than 25% of the patients were aware of the true symptoms of PD.

Regression modeling in this study showed that females were more likely than their male peers to recognize PD problems. This gender‐related result has been continuously reported by many studies and is attributed to females being more aware and concerned about their oral health and appearance (Mamai‐Homata et al., 2016). This was consistent with results reported by previous studies which indicated that females are more obsessed about their esthetic and recognition of periodontal problems (Al‐Johani et al., 2017; Brunsvold et al., 1999).

Undoubtedly, smoking is among the leading risk factors for initiation and progression of PD. Progressive PPD and CAL are well‐recognized adverse effects of smoking (Leite et al., 2018). In addition, current smokers suffer from increasing rate of tooth loss as compared to former smokers or never smokers as demonstrated by a systematic review and meta‐analysis (Souto et al., 2019). The results of the current study were consistent as the smokers showed higher likelihood to report true periodontal CC than never smoker subjects.

It is important to acknowledge that there are universal public attitudes towards dental problems in general and PD in particular, with patients being reluctant to attend the dental clinic unless the disease is painful, esthetically disfiguring or seriously interfering with their masticatory function (Devaraj & Eswar, 2012). The results of our study were consistent with this notion in that awareness about the existence of PD as a problem was positively associated with increasing PPD. Indeed, progressive loss of periodontal tissue support is accompanied by increasing tooth mobility and esthetic problems which potentially alert and motivate the patient to seek periodontal treatment. Further support for these results can be observed through the inverse relation between true periodontal CC and number of missing teeth. This indicated that the patients most probably are not aware that periodontitis, besides dental caries, is among the most common causes of tooth loss (Passarelli et al., 2020); thereby, extraction of the teeth was considered as the best treatment choice. However, awareness about PD would not be the only reason for the tendency to have teeth extracted as the economic factor could play a major role and tooth extraction being the cheapest solution (Bommireddy et al., 2014).

Despite the fact that gingival inflammation starts with color and shape/contour changes, these symptoms develop slowly due to the chronically progressive nature of PD (Kinane et al., 2017). This in turn renders observing these changes a quite difficult task for the patient due to gradual adaptation to the appearance of the gingiva over time. Similarly, the gradual increase in mass of the dental biofilm is further obscured by its translucency in the earlier stages and difficult to detect unless examined by an expert or by using disclosing agents (Fasoulas et al., 2019). These factors could explain the lack of significant association between these two periodontal parameters (PI and GI) and reporting of true periodontal CC.

Similar to other observational studies, this study was only able to determine the association between different factors and reporting of true periodontal CC, rather than the cause‐effect relationship. Additionally, the patients who were recruited were those seeking treatment and suffering from dental/periodontal problems. In addition, the available records for the current study followed an older classification for periodontal disease which limited further clinical interpretations particularly in discriminating health from gingivitis cases. The aforementioned limitations represent selection/recruitment bias that potentially affect the generalizability of the results. However, this bias could be minimized by stratifying the sample according to different independent variables and multivariant analysis. Therefore, conducting prospective surveys is recommended following the 2017 classification system for periodontal diseases and conditions, which would provide more elaborative clinical data for analysis. One of the strengths of this study was its consideration of a large number of records, which provided an indication about the prevalence of reporting true periodontal CC. However, these results cannot be generalized to the whole Iraqi population and further national surveys are required to confirm the results of the current study.

## 5 CONCLUSIONS

The current study concluded that the subjects' identification of true PD symptoms was low and the majority of them reported other CC that were not relevant to PD. Smoking and deterioration of periodontal health with increased destruction of teeth‐supporting tissues, reflected by PPD, were the main factors motivating the patients to report true periodontal CC. In contrast, males and subjects with higher numbers of missing teeth were negatively associated with reporting true periodontal CC. Educational campaigns are required to motivate and educate people about the alarming symptoms of PD and encourage them to seek treatment at earlier stages.

## CONFLICT OF INTEREST

The authors declare no potential conflict of interest.

## AUTHOR CONTRIBUTIONS

Ali A. Abdulkareem and Sarhang S. Gul were responsible for research concept and design. Nada K. Imran collected the data, Ali A. Abdulkareem and Sarhang S. Gul analyzed the results. Rukhosh H. Abdulraheam was responsible for proofreading the final version of the manuscript and critical revision of the article. All authors contributed in writing and approving the final draft of the manuscript.

## Data Availability

The data that support the findings of this study are available from the corresponding author upon reasonable request.
